# Management Patterns and Outcomes of Invasive Mechanical Ventilation in Patients With Cardiogenic Shock

**DOI:** 10.1016/j.jacadv.2025.101916

**Published:** 2025-07-23

**Authors:** Christopher S. Grubb, Grant Tucker, Neda Bionghi, Catherine Chen, Bethany L. Lussier, Corey D. Kershaw, Gregory Ratti, Roma Mehta, Colby R. Ayers, Nicholas S. Hendren, Justin L. Grodin, Jennifer T. Thibodeau, Ann Marie Navar, Maryjane A. Farr, Sandeep R. Das, James A. de Lemos, Eric J. Hall

**Affiliations:** a Parkland Health System, Dallas, Texas, USA; b Division of Cardiology, Department of Internal Medicine, University of Texas Southwestern, Dallas, Texas, USA; c Division of Pulmonary and Critical Care Medicine, Department of Internal Medicine, University of Texas Southwestern, Dallas, Texas, USA

**Keywords:** cardiogenic shock, critical care cardiology, invasive mechanical ventilation, intubation, respiratory failure

## Abstract

**Background:**

Approximately one-half of patients with cardiogenic shock (CS) require invasive mechanical ventilation (IMV). Much of the data regarding IMV management is extrapolated from other populations, and little is known regarding management and outcomes of patients with CS who require IMV.

**Objectives:**

This study aims to provide data on IMV management in a CS-specific cohort.

**Methods:**

Retrospective study of 104 patients treated for CS requiring IMV at an academic safety net hospital from 2017 to 2023. Indications for IMV, ventilator settings, and medications were obtained. Outcomes included in-hospital mortality, survival to extubation, and reintubation.

**Results:**

Reasons for intubation included ongoing cardiac arrest (37%) and hypoxic respiratory failure (32%). Most were on low-level ventilator support 24 hours after intubation (median fraction of inspired oxygen 40% [IQR: 30%-50%], positive end-expiratory pressure 5 cm H_2_O [IQR: 5-8]). Spontaneous breathing trials were delayed in 78%, primarily due to hemodynamic instability (82%). Nonpalliative extubation occurred in 62% after a median of 4.8 days (IQR: 2.3-8.0). Among patients who received temporary mechanical circulatory support (tMCS) (49%) and survived, tMCS was removed before extubation in 98%. Reintubation occurred in 14% within 48 hours, and in-hospital mortality was 41%.

**Conclusions:**

In this cohort, patients were frequently on minimal ventilator support within 24 hours of intubation, yet spontaneous breathing trials and extubation were delayed due to hemodynamic instability. Rates of failed extubation were comparable to other forms of critical illness. Further research is necessary to determine optimal approaches to ventilator liberation in patients with CS, particularly when hemodynamic derangements or tMCS persist in patients who are otherwise candidates for extubation.

Approximately half of all patients hospitalized with cardiogenic shock (CS) in the modern era require invasive mechanical ventilation (IMV).^1^, ^2^, ^3^, ^4^, ^5^, ^6^ Despite this, few data exist regarding management and outcomes of IMV in patients with CS, particularly those receiving both IMV and temporary mechanical circulatory support (tMCS), leaving a knowledge gap that has been recognized as a key research priority in multiple recent publications.^7^, ^8^, ^9^, ^10^, ^11^, ^12^, ^13^

Due to the paucity of CS-specific data, current guidance regarding the management of IMV in patients with CS is extrapolated from other critically ill populations, primarily patients with acute respiratory distress syndrome (ARDS) in the medical intensive care unit (ICU).^7^^,^^11^^,^^14^^,^^15^ However, patients in medical ICUs have key differences in underlying cardiopulmonary pathophysiology and treatment interventions compared to patients with CS. Prior studies in other critically ill populations have highlighted that IMV management approaches may not be applicable across different types of critical illness.^16^^,^^17^

To address this important unmet knowledge gap, we performed a retrospective cohort study of patients with CS who received IMV at our institution to evaluate care patterns surrounding endotracheal intubation, ventilator management, ventilator liberation, and outcomes.

## Methods

### Study design

We performed a single-center retrospective cohort study of patients with CS who received IMV from 2017 through 2023 at Parkland Hospital (PMH), an academic safety-net hospital which is one of the largest urban safety-net hospitals in the United States.^18^ The IMV cohort represents a subset of a larger cohort of patients with CS previously described^19^ (Supplemental Figure 1). At PMH, patients in the cardiac ICU receiving IMV are comanaged by a pulmonary critical care consultant team and a cardiology primary team. During the study period, tMCS was available but patients who required cardiac surgery (including heart transplant or durable left ventricular assist devices) required transfer to an affiliated academic tertiary care hospital. In the years immediately preceding the study period (2015-2017), PMH was a participating hospital in a national quality improvement collaborative led by the Society of Critical Care Medicine (SCCM) to promote protocolized best practices in ICU care.^20^

Study patients were initially identified via an electronic health record (EHR) query to find patients who had undergone a right heart catheterization (RHC), had a left ventricular ejection fraction ≤40%, and had received tMCS or vasoactive medications (vasopressors or inotropes). Data from the EHR were then manually reviewed to confirm a cardiac etiology of shock. Patients who did not experience at least Society of Coronary Angiography and Intervention (SCAI) Stage C or greater CS while intubated were not included, with SCAI staging based on the adapted definitions in Supplemental Table 1.^21^^,^^22^ Patients with prior tracheostomy were excluded. As the goal of this study was to evaluate both management and outcomes of patients receiving IMV, patients intubated for ongoing arrest had to achieve return of spontaneous circulation to be included. For patients with multiple episodes of IMV over the course of a hospitalization, only the first episode was analyzed. This study was approved by the University of Texas at Southwestern Medical Center Institutional Review Board and the Parkland Health and Hospital System Office of Research Administration with a waiver of informed consent.

### Data collection

Data were obtained through a combination of automated and manual extraction. Background characteristics and clinical data were gathered from the medical record. Intubation characteristics were obtained from procedure reports. Race and ethnicity were obtained from the EHR. Illness severity during the first 24 hours of admission was assessed with a modified version of the Sequential Organ Failure Assessment Score using the SaO_2_/fraction of inspired oxygen (FiO_2_) ratio for the respiratory component.^23^ Complications related to intubation were defined as clinical aspiration apparent within 24 hours, or other major complications potentially related to intubation that occurred within 6 hours. Vital signs and SCAI stage were abstracted from the medical record from the following time points: immediately prior to intubation, 1 hour after intubation, 24 hours after intubation, at extubation, and 6 hours after extubation. Ventilator settings were obtained at matching time points while intubated. Delirium was identified from the EHR based on a positive CAM-ICU (Confusion Assessment Method for the ICU) score performed by the bedside nurse when the patient was not comatose.^24^ Indications for intubation, reasons for deferral of spontaneous breathing trials (SBTs), and other clinical decisions were manually abstracted from provider documentation in the EHR. In-hospital mortality (including after transfer to another hospital) was obtained from the PMH EHR and the Dallas Fort Worth Hospital Council database encompassing >90% of hospitals in the North Texas region.^25^ Extubation and reintubation outcomes were not followed after hospital transfer.

For this descriptive study, key features of interest included practices surrounding intubation, medication selection for sedation and analgesia, ventilator management, and ventilator liberation. Key outcomes included duration of intubation, patient outcomes while intubated, in-hospital mortality, and reintubation within 48 hours of extubation.^26^

### Statistical analysis

Study data were collected and stored on REDCap electronic data capture tools hosted and supported by the University of Texas at Southwestern Clinical and Translational Science Award Program.^27^ Categorical data were expressed as a frequency, and continuous data as median with IQR or mean ± SD as appropriate. Fisher exact test was used to perform comparisons between groups. Analyses were performed using SAS software Version 9.4 (SAS Institute Inc) with statistical significance defined as a 2-sided *P* value <0.05.

## Results

### Baseline characteristics

A total of 104 patients met criteria for inclusion. Patient characteristics are shown in Table 1. The median age was 60 years, 71% were male; 38% were of non-Hispanic Black race, 39% of Hispanic ethnicity, and 18% non-Hispanic White. The most common comorbidities were diabetes (59%) and chronic kidney disease (59%), while 13% had a documented history of tobacco use. Most patients (65%) presented with CS due to decompensated heart failure CS, while the remainder (35%) experienced CS due to an acute myocardial infarction (AMI-CS). Invasive hemodynamics are shown in Supplemental Table 2. Almost half (49%) of patients required temporary mechanical support (tMCS) at any time while intubated (40% within 24 hours of intubation).

**Table 1:** Demographics, Comorbidities, and Shock Characteristics (N = 104)

Age (y)	59.7 (52.0-69.0)
Male (%)	71% (74)
Race and ethnicity (%)	
Non-Hispanic Black	38% (40)
Hispanic	39% (41)
Non-Hispanic White	18% (19)
Asian	4% (4)
Comorbidities	
Ejection fraction (%)	34% (25,41)
BMI (kg/m^2^)	27.4 (24.1-32.9)
Chronic kidney disease	59% (61)
End-stage renal disease	8% (8)
COPD	15% (16)
Asthma	3% (3)
OSA	8% (8)
Atrial fibrillation	46% (48)
Stroke	13% (13)
Type 2 diabetes	59% (61)
Tobacco use	13% (14)
Cocaine use	26% (27)
Alcohol use	13% (14)
Type of CS	
AMI-CS	35% (36)
HF-CS	65% (68)
Admission SOFA score	6 (4-8)
Admission lactate (mmol/L)	4.7 (2.2-9.1)
Peak lactate during admission	8.7 (4.7-13.1)
Mechanical circulatory support during admission	
Any device	49% (51)
IABP	40% (43)
Microaxial flow pump	13% (14)
VA-ECMO	3% (3)

Values are median (IQR) or % (n).

AMI = acute myocardial infarction; BMI = body mass index; COPD = chronic obstructive pulmonary disease; CS = cardiogenic shock; HF = heart failure; IABP = intra-aortic balloon pump; OSA = obstructive sleep apnea; SOFA = Sequential Organ Failure Assessment; VA-ECMO = Veno-Arterial Extracorporeal Membrane Oxygenation.

### Intubation indications and practices

**Figure 1:**
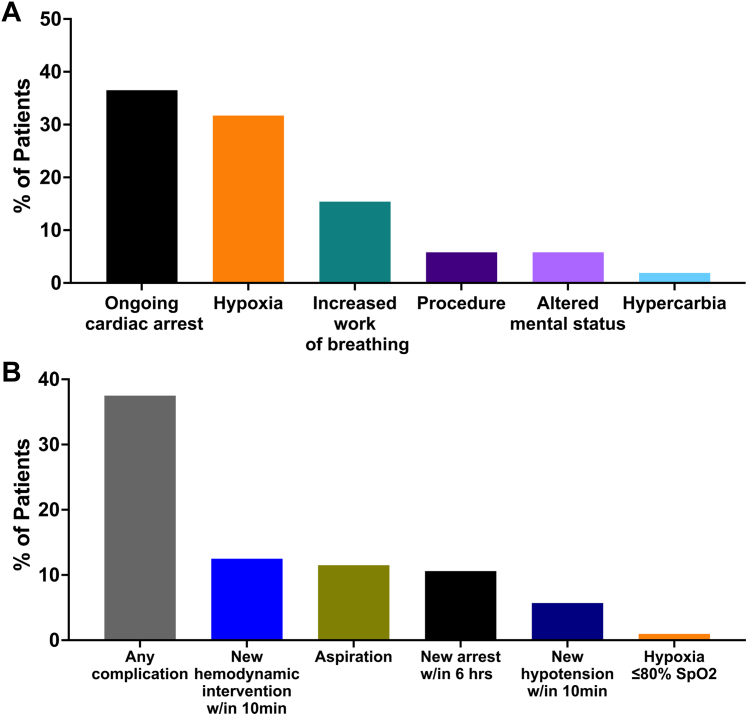
Intubation Indication and Peri-Intubation Complications (A) shows indication for intubation. (B) shows peri-intubation complications.

**Figure 2:**
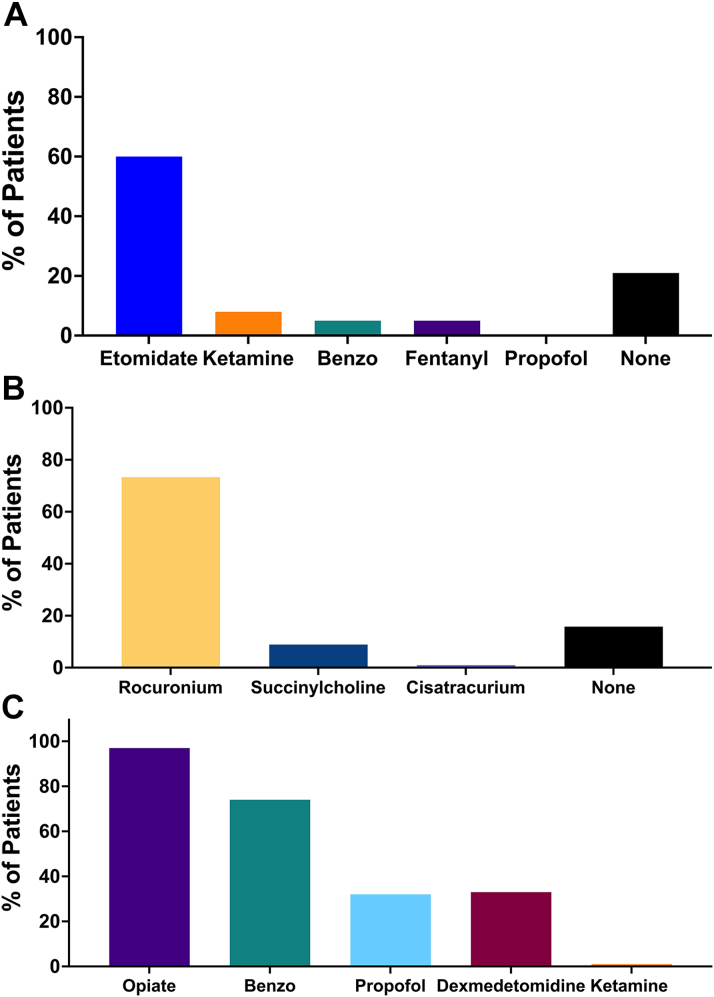
Medications Used for Intubation and Sedation (A) shows induction medications. (B) shows neuromuscular blockade agents. (C) shows medications used for sedation and analgesia during the duration of IMV. IMV = invasive mechanical ventilation.

**Figure 3:**
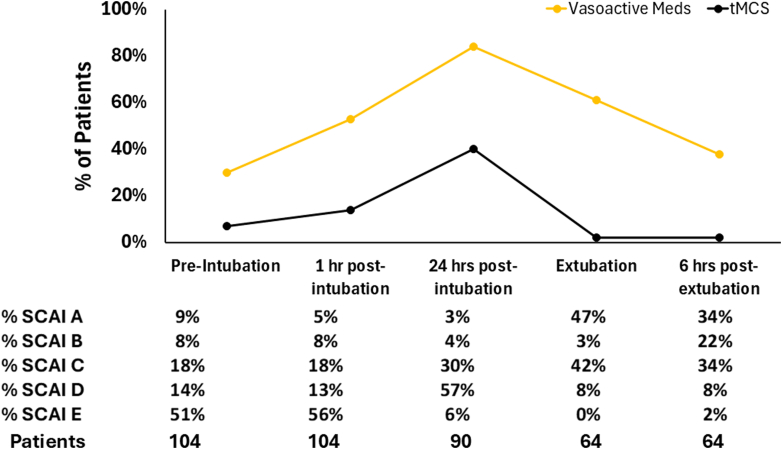
Hemodynamic Support and SCAI Stages While Intubated SCAI = Society for Coronary Angiography and Intervention; tMCS = temporary mechanical circulatory support device.

The most common indications for intubation were ongoing cardiac arrest (37%), hypoxemic respiratory failure (32%), and respiratory distress (15%, Figure 1A). Of those intubated for ongoing cardiac arrest, 31 (82%) were in-hospital arrests and 7 (18%) were out-of-hospital cardiac arrests. Complications following intubation were common with at least one complication occurring in 38% of patients (Figure 1B), most commonly related to hypotension. Etomidate and rocuronium were the most commonly used medications for induction and neuromuscular blockade during intubation, respectively; propofol was not used for induction in any patients (Figures 2A and 2B). Half of patients were in Stage E CS at the time of intubation (Figure 3).

### Ventilator and sedation/analgesia management

**Figure 4:**
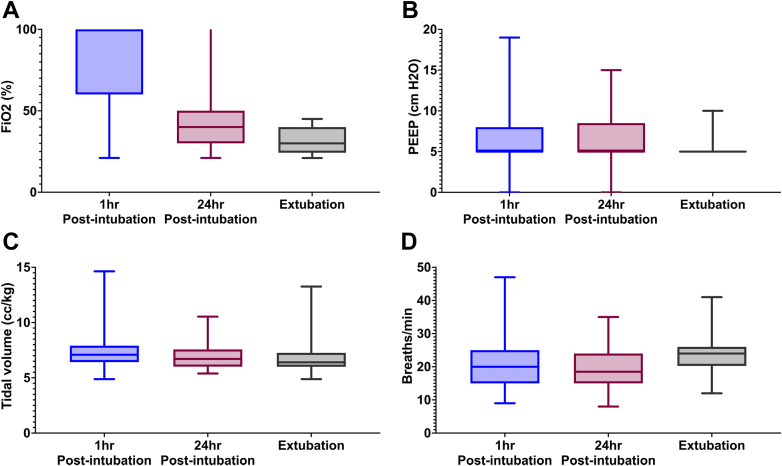
Ventilator Settings Over Time Figure illustrates fraction of inspired oxygen (A), positive end-expiratory pressure (B), tidal volume per ideal body weight (C), and respiratory rate (D) at 1 hour after intubation, 24 hours after intubation, and prior to extubation. FiO_2_ = fraction of inspired oxygen; PEEP = positive end expiratory pressure.

Ventilator settings at 1 hour after intubation, 24 hours after intubation, and prior to extubation are shown in Figure 4. At 24 hours after intubation, most patients were on low-level ventilator support (median FiO_2_ 40%, positive end-expiratory pressure [PEEP] 5 cm H_2_O), with 48% of patients receiving both FiO_2_ ≤40% and PEEP ≤5 cm H_2_O. Patients were managed with low tidal volume ventilation (median 7 cc/kg at all time points) and low PEEP strategies throughout. Volume control mode of ventilation was used in 86% of patients at 24 hours postintubation. Sedation/analgesia medications while on IMV are shown in Figure 2C. Opiate infusions and benzodiazepine infusions were the most commonly used medications, for a median of 5 days (opiate infusions) and 4 days (benzodiazepines) for those who received them. Propofol and dexmedetomidine were each used in almost one-third of patients for a median of 2 days for each. Delirium was common, occurring in 79% of patients.

### Ventilator liberation and outcomes

**Figure 5:**
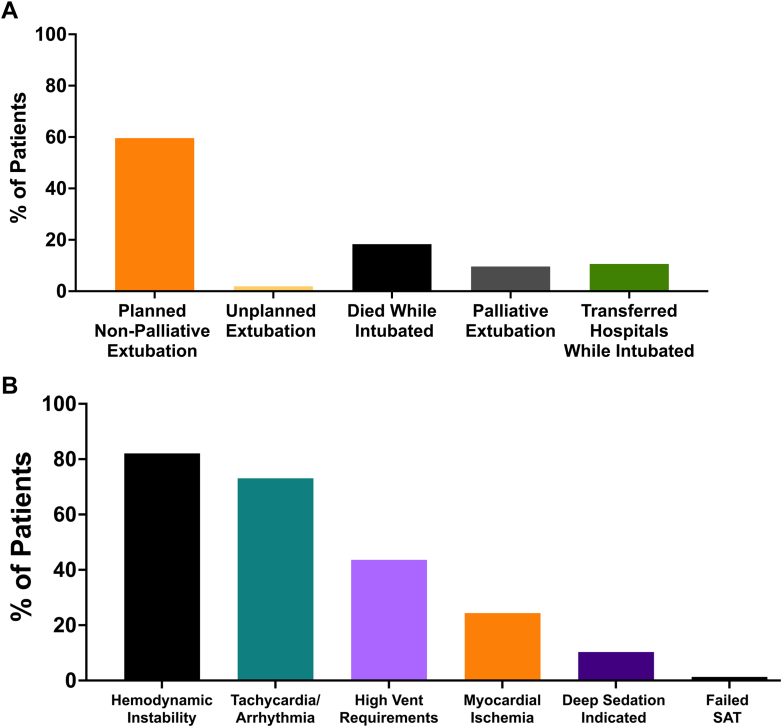
Outcomes and Reasons for Delayed Spontaneous Breathing Trials Figure illustrating outcomes of intubation (A) and reasons for delayed spontaneous breathing trials (B). SAT = spontaneous awakening trial.

A total of 62% of patients survived to planned or unplanned nonpalliative extubation (Figure 5A). These patients were extubated after a median of 4.8 days of IMV (IQR: 2.3-8.0), despite most being on low-level ventilator support by 24 hours. There was no statistical difference in rates of successful extubation between those patients intubated for cardiac arrest vs other indications for intubation (50% vs 68%, respectively, *P* = 0.09). Only 22% of patients had a SBT performed within 48 hours of intubation, with SBTs most commonly documented as delayed due to clinician reporting hemodynamic derangements and/or tachycardia/arrhythmias (Figure 5B). Spontaneous awakening trials (SATs) were likewise delayed, occurring in only 26% of patients within 48 hours. Of 23 patients who received tMCS while intubated and survived to nonpalliative extubation, all but one (2%) had tMCS removed prior to extubation (Figure 3). The vast majority of patients were in Stage A, B, or C CS at the time of extubation; a total of 61% of patients were receiving vasoactive medications just prior to extubation, which decreased to 38% at 6 hours postextubation.

Overall mortality was high, with 41% of patients dying prior to hospital discharge, including 18% who died during their initial episode of IMV (up to 27% including palliative extubation). There was no difference of in-hospital mortality between those patients intubated for cardiac arrest or intubated for other indications (37% vs 44%, respectively; *P* = 0.54). A total of 10% underwent palliative extubation to comfort care and 11% were transferred to another hospital while still intubated (Figure 5A). The most common delayed complications were ventilator-associated pneumonia in 14% and gastrointestinal bleeding in 8%. Reintubation occurred within 48 hours in 14% and 30% at any time during the hospitalization. Of the 19 patients reintubated, 10 were successfully extubated, while 4 died, 2 were palliatively extubated, and 3 required tracheostomy. Most patients were extubated to either nasal cannula or room air (Table 2).

**Table 2:** Intubation Complications and Extubation Outcomes (N = 104)

Complications while intubated	
Delirium	78% (82)
Ventilator-associated pneumonia	14% (15)
Gastrointestinal bleed	8% (8)
Pneumothorax	1% (1)
Oxygen delivery device after extubation	Patients extubated (N = 64)
Nasal cannula	51% (32)
Noninvasive positive pressure ventilation	34% (22)
Other	9% (6)
High-flow nasal cannula	2% (1)
None	5% (3)
Reintubation within 48 hours	14% (9)
Reintubation at any time during hospitalization	30% (19)
Discharged on oxygen	12% (6)

Values are % (n).

## Discussion

In this analysis of patients with CS receiving IMV, we observed several important findings. The majority of patients were on low-level ventilator support within 24 hours of intubation yet remained intubated for a median of almost 4 additional days as SBTs were deferred due to hemodynamic derangements. Removal of tMCS was clearly prioritized over ventilator liberation. Benzodiazepine and opiate infusions were the primary form of sedation/analgesia, with propofol and dexmedetomidine each used in approximately one-third of patients. Finally, failed extubation occurred in 14% of patients.

Heart failure CS was the most common etiology of CS, similar to other published cohorts.^1^^,^^28^^,^^29^ This cohort of patients with CS requiring IMV demonstrated high illness severity, with over half of patients reaching SCAI Stage E at some point while receiving IMV. Concordant with this, median admission lactate was 4.7 mmol/L, while the median peak lactate while hospitalized was 8.7 mmol/L. Both lactate values and in-hospital mortality were higher in our population than in CS cohorts reported from the Cardiac Critical Care Trials Network and the Cardiogenic Shock Working Group, likely due to selection for only patients requiring IMV in our cohort.^1^^,^^28^ This highlights that patients with CS who require IMV represent a high-risk subset in need of advances in care.

Indications for IMV in this cohort were varied. Primary respiratory failure (34%) and increased work of breathing (15%) were common, but many patients were intubated for nonrespiratory indications (eg, cardiac arrest). It is important to note that of those intubated for cardiac arrest, the majority were in-hospital cardiac arrests. However, 7 patients were intubated for out-of-hospital arrests, which carries a far worse prognosis.^30^^,^^31^ Peri-intubation complications were frequent (38%), with the most common being an increase in hemodynamic support or postintubation cardiac arrest. The rate of peri-intubation complications was largely similar, however, to that reported in the INTUBE (International Observational Study to Understand the Impact and Best Practices of Airway Management in Critically Ill Patients) prospective registry of airway management. The rate of early cardiac arrest after intubation was similar in our cohort to the INTUBE registry as well (3% in both cohorts).^32^ In our cohort, half were at SCAI Stage E at the time of intubation. A secondary analysis of the TRIUMPH (Tilarginine Acetate Injection in a Randomized International Study in Unstable MI Patients With Cardiogenic Shock) trial showed that among patients with CS, earlier initiation of IMV was associated with lower mortality than delayed intubation.^33^

Selection of medications for induction and sedation in patients with CS is challenging since few data are available and all sedating medications can result in at least some degree of hypotension. In the INTUBE registry, propofol was the most commonly used induction agent, followed by midazolam and etomidate.^32^ Propofol is generally considered to have the greatest negative inotropic effect of available medications for induction, with etomidate among the most hemodynamically neutral.^7^^,^^34^ In our population, etomidate was by far the most commonly used induction agent, with no use of propofol. Recent data from the Vizient administrative database in patients with AMI who required intubation suggested an association between propofol use for induction and lower mortality when compared to etomidate in a propensity-matched analysis.^34^ This study was published after our study period, and it remains to be seen how it will impact clinical practice. Moreover, residual confounding by indication makes observational data challenging to interpret. Ultimately randomized trials will be necessary to provide further insight into optimal medication selection in this high-risk population.

For sedation and analgesia while intubated, the SCCM recommends propofol or dexmedetomidine over benzodiazepines for sedation to minimize the incidence of delirium.^35^ In contrast, an AHA (American Heart Association) Scientific Statement recommends avoiding propofol and dexmedetomidine in CS given the risk of hypotension.^14^ In our cohort, 97% of patients received an opiate infusion and 73% a benzodiazepine infusion, each for a median of 5 and 4 days, respectively. In addition, one-third of patients received propofol and one-third dexmedetomidine at some point while intubated (for a median of approximately 2 days). It is important to note PMH restricts dexmedetomidine usage to within 48 hours of expected extubation. In effect, this sedation/analgesia strategy aimed first to preserve hemodynamics at the expense of increased risk of delirium as both opiates and benzodiazepines are associated with higher rates of delirium than other agents.^36^ The rate of delirium in our cohort (79%) was higher than observed in prior cohorts of cardiac intensive care unit (CICU) and CS patients (∼20% to 25%).^37^, ^38^, ^39^ However, the rate of delirium in our cohort was similar to that seen in a medical ICU cohort of patients who all received IMV.^24^ It is also important to note that delirium in this study was defined by CAM-ICU score, and differentiation between true delirium or other acute brain injury from events such as cardiac arrest is challenging. Given the association between delirium and long-term cognitive impairment observed in other ICU populations, future studies of sedation strategies for patients with CS receiving IMV which include long-term outcomes will be necessary to inform care strategies in the CICU.^40^

After initiation of IMV, patients were initially placed on relatively high ventilator support, but by 24 hours, almost half of patients had been weaned to “minimal vent settings,” conservatively defined as FiO_2_ ≤40% and PEEP ≤5 mm Hg. The low ventilator requirements suggest pulmonary edema and respiratory insufficiency were generally not the reason for continued IMV support. Additionally, median tidal volume was 7 cc/kg, slightly above the traditional 6 cc/kg used for low tidal volume ventilation. While this cohort was managed with lower tidal volumes and PEEP, there are no data suggesting improvement in clinical outcomes with this strategy in CS patients. The literature surrounding low tidal volume ventilation is primarily taken from ARDS patients, and it is unclear if strict tidal volume control or a target driving pressure is associated with similar mortality improvements in CS patients as it is in ARDS.^17^ This cohort was also predominantly left ventricular or mixed biventricular failure, so the potential harm of higher PEEP strategy associated with right ventricular dysfunction would be less likely. Further work is needed to assess if low tidal volume or lower PEEP strategies impact clinical outcomes in this patient population.

Both the SCCM and AHA recommend routine protocolized use of SATs and SBTs to facilitate extubation based on data that these interventions improve all-cause mortality in intubated patients.^14^^,^^41^^,^^42^ Patients must pass a “safety screen” before being eligible for these tests. Criteria for SBT safety are not standardized, but typically include factors such as being on low ventilator settings and relative hemodynamic stability.^42^^,^^43^ In some medical ICU literature, milrinone (at any dose) is classified as a screen failure.^42^ Failing SBT screens for inotrope use is likely not appropriate in patients with CS. Even in the absence of SBT criteria, patients should receive SATs unless contraindicated due to factors such as the patient receiving paralytic agents or in severe shock (3-5 pressors in medical intensive care unit literature).^15^^,^^42^ It is important to note that tMCS is not included in these recommendations. There are few data on weaning IMV in the setting of inotropic or mechanical circulatory support,^15^ but data from the medical ICU increasingly suggest that extubation on low-dose vasopressors is reasonable.^44^ The definition of how much support qualifies as “hemodynamically unstable” needs further investigation in the modern era of CS.

Despite most patients being on minimal ventilator support within 24 hours after intubation and ICU protocols for routine SATs/SBTs, only 26% of patients received an SAT and 22% an SBT within 48 hours after intubation. The most commonly cited reason for delay documented in the EHR was hemodynamic instability. Weaning of hemodynamic support, particularly tMCS, was prioritized over ventilator liberation, as only a single patient was extubated while receiving tMCS. As a result, the median time to extubation in our cohort was almost 5 days. The 14% reintubation rate is consistent with the expected 10% to 20% reintubation rates reported in the literature from general ICU populations, although it is higher than was described in CICU patients in the Cardiac Critical Care Trials Network of 7.6%, the majority of whom did not have CS.^6^^,^^45^ Patients with cardiac disease are known to be at higher risk of reintubation, so the relatively low reintubation rate in our study raises the question of whether IMV could safely have been discontinued earlier.^12^^,^^45^ Additionally, most patients were extubated to room air or nasal cannula, non-invasive positive pressure ventilation, suggesting respiratory insufficiency was not the primary driver of remaining intubated. It is possible that using different criteria to screen for the safety of SATs/SBTs may be necessary to accelerate extubation for patients with CS, thereby limiting complications of extended duration of IMV such as increased risk of delirium and immobility.

Half of patients in our study received both tMCS and IMV, a population in whom very little data exist regarding management and outcomes. There are more data regarding ventilator management in VA-ECMO (Veno-Arterial Extracorporeal Membrane Oxygenation) patients, but this does not generalize easily to broader populations with CS such as ours.^46^ It may be that hemodynamic support from inotropic agents, tMCS, or both would facilitate earlier successful extubation, but this needs further study. These questions highlight the difficulty of applying the medical ICU data to the CS patient and suggest the need to further evaluate definitions of “hemodynamic instability” when considering weaning of IMV.

### Study Limitations

This study is a retrospective single-center cohort study with inherent limitations. Data from this academic safety-net hospital may not be generalizable to all practice settings. For instance, VA-ECMO use was minimal during the study period, and heart replacement therapies such as durable left ventricular assist device or transplant are limited in availability and required transfer to another facility. Availability of these therapies may differ widely between institutions. This study only included patients who had RHC to document hemodynamics during the admission. This may lead to selection bias by excluding patients who had CS but did not receive RHC, although data from multicenter cohorts suggests that pulmonary artery catheter use is common nationally in patients with SCAI D or E CS, the subgroup that comprised the majority of our population.^47^^,^^48^ Additionally, many patients in this cohort suffered cardiac arrest, primarily in-hospital arrest. Our patient population, representing patients with CS treated at a safety-net hospital who had a high incidence of cardiac arrest and who were eligible for a RHC, may have important differences from patients treated at other hospitals, and this limitation should be considered when attempting to generalize these data. Data were all manually reviewed for accuracy, but clinical reasoning documented in the EHR may not reflect full decision-making regarding extubation practices. This study is designed to provide insight into current practice patterns but is not designed or powered to compare specific strategies.

## Conclusions

Data on management of IMV in patients with CS are very limited. In this study which provides granular data on IMV in a cohort of patients with CS, we observed that hemodynamic factors were the primary reason for delayed extubation, as most patients were on low-level ventilator support within 24 hours of intubation but remained intubated for a median of 4.8 days (Central Illustration). Weaning of tMCS was prioritized over ventilator liberation. These data suggest further study is necessary to determine optimal strategies for ventilator liberation in patients with CS, where in contrast to other critically ill populations; hemodynamic derangements rather than respiratory insufficiency are the primary barrier to extubation. Ultimately, multicenter, prospective cohort studies will be necessary to understand ventilatory practices and outcomes in patients with CS.

Perspectives **COMPENTENCY IN PATIENT CARE:** This study illustrates the diversity and complexity of the CS population requiring invasive mechanical support. It shows how management strategy is frequently based on literature intended for other patient populations, and this may or may not be applicable to the CS population.

**TRANSLATIONAL OUTLOOK:** Most importantly, it highlights the need for further work in understanding the correct approach to IMV in the CS patient.

**Central Illustration:**
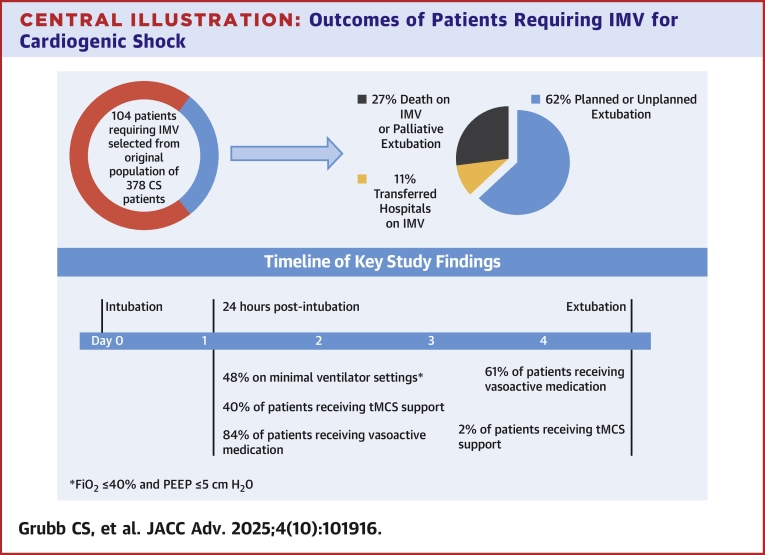
Outcomes of Patients Requiring IMV for Cardiogenic Shock Ventilation outcomes and required support by time point. Abbreviations as in Figures 2, 3, and 4.

## Funding support and author disclosures

This work was supported by 10.13039/100016220CTSA Grant Number UL1 TR003163 from the 10.13039/100006108National Center for Advancing Translational Science (NCATS), a component of the 10.13039/100000002National Institutes of Health (NIH). Dr Hall is supported by the 10.13039/100000050NIH/National Heart, Lung, and Blood Institute through grant 5T32HL125247-8. Dr Hendren has received research funding from the 10.13039/100000968American Heart Association, Tosoh, Inc, and TriCog health. Dr Grodin has received personal fees from 10.13039/100004319Pfizer, 10.13039/100006400Alnylam, Eidos/BridgeBio, AstraZeneca, Alexion, Lumanity, Novo Nordisk, Ultromics, and Intellia and grant support from NHLBI, Pfizer, Eidos/BridgeBio, and Texas Health Resources Clinical Scholars Fund. All other authors have reported that they have no relationships relevant to the contents of this paper to disclose.
