# Supplementary material for: Management Patterns and Outcomes of Invasive Mechanical Ventilation in Patients With Cardiogenic Shock
Source: JACC Adv. 2025 Jul 23;4(10):101916. doi: 10.1016/j.jacadv.2025.101916 (PMC12541230; doi:10.1016/j.jacadv.2025.101916)
Supplement: Supplemental Material [file mmc1.docx]

Supplemental Figure 1: Consort Diagram


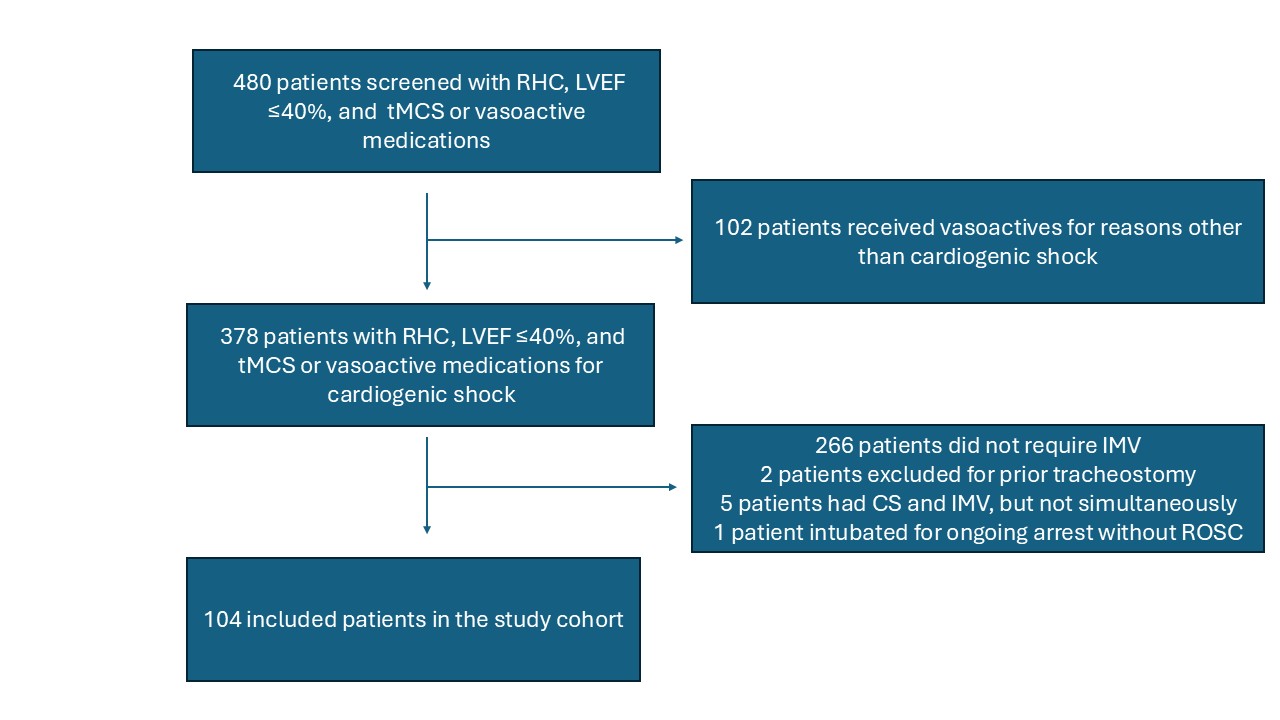


Supplemental Table 1: Applied Definitions of SCAI Stages

| **SCAI Stage** | Definition |
| --- | --- |
| **A** | None of the following criteria |
| **B** | HR>100 or SBP<90 mmHg on 2 separate measurements 15+ minutes apart |
| **C** | 1 vasoactive medication or 1 tMCS device |
| **D** | 2+ vasoactives or vasoactive+tMCS |
| **E** | Any of the following criteria:   - - SCAI C/D criteria + (lactate>8mmol/L or bicarbonate<14mmol/L)   - VA ECMO   - In-hospital cardiac arrest   - Hemodynamic collapse requiring use of push-dose vasoactive medications |

HR: Heart Rate; SCAI: Society of Coronary Angiography and Intervention; tMCS: Temporary Mechanical Circulatory Support; VA ECMO: Veno-arterial Extracorporeal Membrane Oxygenation

Supplemental Table 2: Invasive Hemodynamics

|  | Pre-Intubation (N=22 for RA, N = 13-19 for other measures) | One Hour After Intubation (N=47 for RA, N=37-44 for other measures) | 24 Hours After Intubation (N=58 for RA, N=27-52 for other measures) | Prior to Extubation (N=37 for RA, N=12-22 for other measures) |
| --- | --- | --- | --- | --- |
| Right Atrium (RA) (mmHg) | 12 (6.8,14.8) | 14 (10,19) | 10 (8,14) | 8 (6,12) |
| Pulmonary Artery Systolic (PAs) (mmHg) | 47 (40,57) | 45 (37,57) | 38 (32,44) | 44 (32,60) |
| Pulmonary Artery Diastolic (PAd) (mmHg) | 25 (21,31) | 25 (19,32) | 21 (16,26) | 22 (19,30) |
| Pulmonary Artery Mean (PAm) (mmHg) | 34 (26,41) | 33 (26,39) | 27 (24,32) | 30 (23,37) |
| Pulmonary Capillary Wedge Pressure (PCWP) (mmHg) | 21 (15,29) | 21 (16,30) | 21 (15,28) | 18 (14,25) |
| Cardiac Output (CO) (L/min) | 3.3 (2.5,3.7) | 3.8 (2.8,4.6) | 3.9 (3.3,4.5) | 4.7 (3.9,5.7) |
| Cardiac Index (CI) (L/min/m^2^) | 1.6 (1.5,1.8) | 1.9 (1.6,2.4) | 2 (1.8,2.4) | 2.7 (2.1,3.0) |
